# Supplementary material for: Mechanisms governing the pioneering and redistribution capabilities of the non-classical pioneer PU.1
Source: Nat Commun. 2020 Jan 21;11:402. doi: 10.1038/s41467-019-13960-2 (PMC6972792; doi:10.1038/s41467-019-13960-2)
Supplement: Supplementary file 7 — Source data [file 41467_2019_13960_MOESM7_ESM.zip › Source_Data/FigureS2/FigureS2B_MotifScanOutput/KmeansCluster7/homerResults/motif16.similar.html]

motif16

## Information for motif16

C
G
T
A
A
G
T
C
C
G
T
A
A
C
T
G
A
T
C
G
C
G
T
A
C
G
T
A
  
Reverse Opposite:  

A
C
G
T
A
C
G
T
A
T
G
C
A
G
T
C
A
C
G
T
A
C
T
G
A
C
G
T
  

|  |  |
| --- | --- |
| p-value: | 1e-67 |
| log p-value: | -1.550e+02 |
| Information Content per bp: | 1.954 |
| Number of Target Sequences with motif | 698.0 |
| Percentage of Target Sequences with motif | 21.74% |
| Number of Background Sequences with motif | 4653.5 |
| Percentage of Background Sequences with motif | 10.64% |
| Average Position of motif in Targets | 100.0 +/- 52.7bp |
| Average Position of motif in Background | 101.8 +/- 71.2bp |
| Strand Bias (log2 ratio + to - strand density) | -0.1 |
| Multiplicity (# of sites on avg that occur together) | 1.09 |
| Motif File: | file (matrix) reverse opposite |

### Similar de novo motifs found

|  |  |  |  |  |  |  |  |
| --- | --- | --- | --- | --- | --- | --- | --- |
| Rank | Match Score | Redundant Motif | P-value | log P-value | % of Targets | % of Background | Motif file |
| 1 | 0.832 | A C T G A T C G C G A T C A G T A G T C A G T C G A C T C T A G A G C T C G A T A C G T A C G T A G C T | 1e-54 | -125.665462 | 7.98% | 2.36% | motif file (matrix) |
